# Intraoperative Whole Sentinel Lymph Node Analysis Using the OSNA Assay in Early-Stage Cervical Cancer: A Comparative Study

**DOI:** 10.3390/cancers17111753

**Published:** 2025-05-23

**Authors:** Shinichi Togami, Nozomi Furuzono, Mika Fukuda, Hiroaki Kobayashi

**Affiliations:** Department of Obstetrics and Gynecology, Faculty of Medicine, Kagoshima University, Sakuragaoka 8-35-1, Kagoshima 890-8520, Japan; k6641707@kadai.jp (N.F.); sakura.mika1014@icloud.com (M.F.);

**Keywords:** cervical cancer, micrometastasis, one-step nucleic acid amplification, sentinel lymph node

## Abstract

**Objective:** This study aimed to evaluate the reliability and clinical utility of intraoperative sentinel lymph node (SN) metastasis diagnosis using the one-step nucleic acid amplification (OSNA) assay in early-stage cervical cancer by comparing its accuracy with conventional histopathological examination. **Methods:** A retrospective analysis was conducted on 163 patients who underwent SN biopsy at Kagoshima University Hospital between April 2014 and December 2024. This study included 50 and 113 patients in the OSNA assay and histopathological diagnosis groups, respectively. The OSNA assay quantified cytokeratin 19 (CK19) mRNA levels to determine SN metastasis. The surgical outcomes, SN metastasis detection rates, and non-SN metastasis status were compared between the two diagnostic methods. **Results:** The SN metastasis detection rate was significantly higher in the OSNA group (12%) than in the pathology group (3%) (*p* < 0.05). The OSNA assay identified only micrometastases (+) among the positive cases, whereas histopathological diagnosis detected both macrometastases and micrometastases. No non-SN metastases were observed in any of the SN-positive cases, and no significant differences were observed in the recurrence rates between the two groups. **Conclusions:** The OSNA assay demonstrated a higher SN metastasis detection rate than conventional pathology and demonstrated superior sensitivity in identifying micrometastases. These findings suggest that intraoperative OSNA-based SN assessment in cervical cancer could improve staging accuracy and potentially reduce the need for systematic lymphadenectomy. However, further prospective studies are warranted to confirm these findings and establish clinical guidelines.

## Simple Summary

Reports on cervical cancer are limited, and, to our knowledge, no studies have evaluated intraoperative diagnosis using whole sentinel lymph node analysis with the one-step nucleic acid amplification (OSNA) assay. This study demonstrated that intraoperative diagnosis using whole sentinel lymph nodes with the OSNA assay provides diagnostic accuracy comparable to conventional intraoperative pathological examination for lymph node metastasis. Furthermore, consistent with findings in other cancer types, the OSNA assay showed a higher sensitivity in detecting micrometastases compared to conventional pathological examination. These findings suggest that OSNA-based intraoperative assessment for sentinel lymph node metastasis may become more widely adopted, alleviating the workload of pathologists and pathology technicians while enabling the detection of micrometastases with greater precision.

## 1. Introduction

In cervical cancer, the presence or absence of pelvic lymph node metastasis is a critical prognostic factor [1]; therefore, pelvic lymphadenectomy (PLA) is considered a standard surgical procedure [2]. However, complications associated with PLA, such as vascular and nerve injury, lower limb lymphedema, lymphocele formation, and bowel obstruction, often result in a decline in the patient’s quality of life, posing significant clinical challenges [3,4].

The sentinel lymph node (SN) is defined as the first lymph node that receives lymphatic drainage from the primary tumor, making it the initial site for lymphatic metastasis. According to the SN theory, if no metastasis is detected in the SN, metastasis in other lymph nodes is unlikely. SN biopsy for cervical cancer not only reduces the incidence of lymphedema and lymphocele, but also facilitates accurate metastatic diagnosis [5]. Consequently, SN biopsy has recently been established as a standard treatment in many Western countries [6,7]. Micrometastases (MIC), defined as tumor deposits measuring 0.2–2 mm, have emerged as a clinically significant finding in SN biopsies for cervical cancer. Recent evidence suggests that the presence of MIC is associated with a significantly worse prognosis compared to node-negative cases, with decreased disease-free and overall survival—comparable to that seen with macrometastases [8]. These findings underscore the potential clinical relevance of intraoperative detection of MIC, particularly using molecular methods such as the OSNA assay.

Currently, SN metastasis is predominantly assessed using histopathological diagnosis. However, the one-step nucleic acid amplification (OSNA) assay, a molecular diagnostic method for lymph node analysis, has garnered increasing attention. The OSNA assay employs specialized gene amplification devices and reagents to amplify and detect cytokeratin 19 (CK19) mRNA in solubilized lymph nodes, enabling the identification of cancer metastasis. Unlike reverse transcription polymerase chain-reaction-based molecular biology approaches that require nucleic acid extraction and purification [9], the OSNA assay enables one-step amplification and provides results within 30–40 min.

Comparative studies of the diagnostic performance of the OSNA assay and conventional histopathological analysis have been conducted for various cancers, including breast cancer, with most studies reporting no significant differences between the two methods [10,11,12,13]. Recently, similar comparative studies on cervical cancer have also confirmed similar diagnostic performance of the OSNA assay and histopathological examination [10,14,15]. However, in these studies, individual lymph nodes were divided for analysis, with part of the node being examined by histopathology and the other part analyzed using the OSNA assay. These studies focused on the concordance rates but did not evaluate the reliability of diagnosing metastasis using the OSNA assay alone for the entire lymph node in clinical practice.

This study aimed to evaluate the reliability and clinical utility of intraoperative metastasis diagnosis using the OSNA assay on whole SNs in cervical cancer and compare its accuracy with that of conventional histopathological examination.

## 2. Materials and Methods

### 2.1. Patients

This study included patients who underwent SN biopsy at Kagoshima University Hospital between April 2014 and December 2024 with a preoperative diagnosis of early-stage cervical cancer. The eligibility criteria included a histological diagnosis of cervical cancer (excluding rare histological subtypes), a maximum tumor diameter of ≤3 cm, as confirmed by preoperative imaging, and no evidence of distant metastasis or lymph node enlargement. Patients in whom SNs could not be identified bilaterally during surgery were excluded. This study was approved by the Institutional Review Board of Kagoshima University Hospital (Approval No. 200154-1), and written informed consent was obtained from all of the patients prior to surgery. Patient information was collected retrospectively from electronic medical records. In accordance with the journal’s guidelines, we will provide our data for independent analysis by a team selected by the Editorial Team for the purposes of additional data analysis or for the reproducibility of this study in other centers if such is requested.

### 2.2. SN Identification

SNs were identified using a hybrid method combining the radioisotope technique and the indocyanine green (ICG) method, as previously described [5]. Radioisotope SN mapping involved the peritumoral injection of technetium-labeled phytate prior to surgery, followed by lymphoscintigraphy and single-photon emission computed tomography/computed tomography to identify the location of the SNs. For ICG mapping, ICG was injected into the peritumoral area immediately before surgery, and SNs were visualized intraoperatively using a near-infrared fluorescence laparoscopic camera system or the Firefly system of the Da Vinci Xi platform. Identified SNs were excised for further analysis. Systematic PLA was omitted in cases where intraoperative diagnosis determined that the SNs showed no metastasis; in addition, in cases with positive SN metastasis, systematic PLA was performed.

### 2.3. Histopathological SN Examination

Excised SNs were sectioned transversely at 2 mm intervals and submitted for rapid histopathological examination with hematoxylin and eosin staining. Ultra-staging of SNs was not performed in this study.

### 2.4. OSNA Assay for SN

Starting in February 2021, OSNA-assay-based SN metastasis diagnosis was implemented for cases where the primary tumor demonstrated positive CK19 expression. For CK19-negative cases, conventional histopathological diagnosis was performed. For OSNA analysis, excised SNs were bisected along their largest cross-section, and imprint cytology was performed on the cut surface. Subsequently, the whole SN was subjected to OSNA assay for detecting any metastasis. The OSNA assay was performed using the same methodology reported in previous studies [14]. To minimize RNA degradation, SNs were processed immediately after excision. Each SN slice was homogenized on ice in 4 mL of glycine buffer (200 mmol/L glycine, 20% dimethyl sulfoxide, 5% detergent; pH 3.5) and centrifuged at 10,000× *g* for 60 s at room temperature. The intermediate layer of the solubilized lymph node solution was then collected. Using this solution, CK19 mRNA was amplified via the reverse transcription-loop-mediated isothermal amplification method with six primers, utilizing LS93R and a specialized device (RD-200). To verify the RNA integrity, β-actin mRNA levels were also measured using the OSNA system, and the results were generated in approximately 30–40 min. Based on prior research [16], SNs were classified as metastasis-positive if the CK19 mRNA levels were ≥250 cCP/μL and metastasis-negative if <250 cCP/μL. The positive samples were further divided into micrometastasis (+) for values between 250 and 5000 cCP/μL and macrometastasis (++) for values exceeding 5000 cCP/μL, as described in previous studies [17]. No specific cutoff was defined for the isolated tumor cells.

## 3. Results

A total of 163 patients were analyzed, comprising 50 patients in the OSNA assay group and 113 patients in the histopathological diagnosis group. Table 1 presents the clinicopathological characteristics of these patients. The median age and body mass index in the OSNA group were 42 years (range: 29–78) and 21.9 (range: 17.1–34.8), respectively, while those in the pathology group were 39 years (range: 20–74) and 21.6 (range: 16.5–50.4), respectivley, with no statistically significant differences between the two groups. In the OSNA group, laparoscopy was the most frequently performed surgical approach (29 patients, 58%), whereas, in the pathology group, laparotomy was predominant (71 patients, 63%), showing a significant difference between the groups (*p* = 0.0017). The final histopathological subtype was predominantly squamous cell carcinoma in both groups, with no significant difference observed.

The incidence of SN metastasis positivity was significantly higher in the OSNA group (6 patients, 12%) than in the pathology group (4 patients, 3%). All six SN-metastasis-positive cases in the OSNA group were classified as micrometastases (+). In contrast, in the pathology group, three of four SN-metastasis-positive patients had macrometastases, while one patient had micrometastasis. Recurrence was not observed in the OSNA group, whereas four patients (4%) in the pathology group experienced recurrence; however, this difference was not statistically significant.

Further analysis of the clinicopathological characteristics of the SN-metastasis-positive cases is presented in Table 2. In the OSNA group, three of six SN-metastasis-positive patients had cervical stromal invasion extending beyond one-half of the cervical thickness, and four patients exhibited lymphovascular space invasion (LVSI). The OSNA assay detected micrometastases (+) in all six cases, with stamped smear cytology showing negative results in five cases and suspicious findings in one case. None of the six SN-metastasis-positive patients had metastases in the non-SN lymph nodes retrieved from systematic PLA, and no recurrence was observed in any of these patients. Similarly, in the pathology group, all four SN-metastasis-positive patients underwent systematic PLA. Among them, three had cervical stromal invasion extending beyond one-half of the cervical thickness, and LVSI was present in all cases. No metastases were detected in the non-SN lymph nodes, and no recurrence was observed in these patients.

## 4. Discussion

### 4.1. Summary of Main Results

In this study, we evaluated the reliability and clinical utility of intraoperative metastasis diagnosis using the OSNA assay on whole SNs in early-stage cervical cancer and compared its accuracy with conventional histopathological examination. The results demonstrated that the OSNA group had a significantly higher SN metastasis detection rate than the pathology group. Furthermore, the OSNA assay exhibited a greater capability for detecting micrometastases (+) than conventional pathology.

### 4.2. Results in the Context of the Published Literature

The OSNA assay has a reported diagnostic accuracy comparable to that of histopathological examination for lymph node metastasis across various cancer types [18,19,20,21]; however, its application in cervical cancer remains limited. The first study evaluating the accuracy of OSNA assay in cervical cancer was reported by Okamoto et al., who established a cutoff value of 250 copies/μL, concluding that the OSNA assay provided equivalent diagnostic performance to the 2 mm interval histopathological examination [22]. Our previous single-institution study evaluating the diagnostic performance of the OSNA assay in cervical cancer reported a concordance rate, sensitivity, and negative predictive value (NPV) of 80%, 97.7%, and 97.7%, respectively, indicating diagnostic accuracy comparable to that of histopathological examination [14]. Furthermore, a multicenter study assessing the diagnostic performance of the OSNA assay in cervical cancer reported a concordance rate, sensitivity, and NPV of 96.4%, 90.3%, and 97.6%, respectively, suggesting that the OSNA assay has the potential to serve as a useful intraoperative diagnostic tool for lymph node metastasis in cervical cancer [10].

In the present study, intraoperative metastasis diagnosis was performed using the OSNA assay on all resected whole SNs in early-stage cervical cancer. The results demonstrated that the SN metastasis detection rate was significantly higher in the OSNA group (6 patients, 12%) than in the pathology group (4 patients, 3%). Previous studies on breast cancer have reported that the OSNA assay enables the intraoperative identification of a greater number of lymph node metastases than conventional histopathology [23]. Similarly, in endometrial cancer, the ENDO-OSNA trial reported that the OSNA assay identified 10.9% more lymph node metastases than conventional pathology [24]. Based on these findings, applying the OSNA assay for whole SN metastasis diagnosis in cervical cancer may also enable the detection of a higher number of SN metastases intraoperatively, which may contribute to more accurate postoperative decision making regarding adjuvant therapy, thereby improving prognostic stratification for patients with cervical cancer.

In the present study, six cases in the OSNA group were diagnosed with SN metastases, all of which were classified as micrometastases (+). In contrast, among the four SN-metastasis-positive cases in the pathology group, only one patient had micrometastasis, while the remaining three had macrometastases. These findings suggest that the OSNA assay is superior to conventional histopathology in detecting small-volume metastases, such as micrometastases. Previous studies in breast cancer comparing the intraoperative SN metastasis detection capabilities of the OSNA assay with conventional pathology have reported that the OSNA assay significantly enhances the identification of micrometastases [25,26]. Although limited to a cohort of 18 cases, an OSNA-based study in cervical cancer demonstrated that the assay successfully identified micrometastases in six patients (33.3%) during intraoperative assessment [27]. Similarly, in lung cancer, the OSNA assay has been reported to have a higher detection rate for micrometastases than conventional pathology [28]. Furthermore, a meta-analysis evaluating intraoperative OSNA-based lymph node metastasis detection across multiple malignancies, including gynecological, head and neck, thyroid, colorectal, gastric, and lung cancers, has confirmed a high diagnostic performance of the OSNA assay in detecting lymph node metastases [29]. While the clinical significance of micrometastases varies across various cancer types, the ability to detect even small-volume metastases in cervical cancer is particularly valuable, as it ensures more accurate staging and therapeutic decision making.

In the context of SN biopsy, assessing the likelihood of non-SN metastases in patients with SN-positive disease is of paramount clinical importance. If micrometastatic SN involvement is associated with a negligible risk of non-SN metastasis, patients with SN micrometastases may be spared systematic lymphadenectomy, thereby reducing surgical morbidity. Ohi et al., in a study on whole SN analysis in breast cancer, identified an OSNA-based SN metastasis copy number exceeding 5000 cCP/μL (macrometastasis ++) to be the most significant predictor of non-SN metastasis [30]. Similarly, Monterossi et al. investigated non-SN metastases in endometrial cancer and reported that all cases of non-SN metastasis were associated with macrometastatic (++) SN involvement, whereas none of the cases with SN micrometastases exhibited non-SN metastases [31].

### 4.3. Strengths and Weaknesses

In the present study, all six OSNA-positive cases were classified as micrometastases, and none of these patients exhibited non-SN metastases. Although the sample size remains limited, the findings of this study suggest that, in cervical cancer, SN micrometastasis alone may be associated with an extremely low likelihood of non-SN involvement. This could have important clinical implications in supporting a more conservative surgical approach, potentially omitting systematic lymphadenectomy in selected patients.

This study has several limitations. First, the retrospective design inherently carries the risk of selection and information bias. Second, the number of patients evaluated with the OSNA assay was relatively small, which may limit the generalizability of our findings. Third, this study was conducted at a single institution, and, therefore, the results may not be broadly applicable to other settings. Fourth, long-term oncologic outcomes were not assessed, making it difficult to draw conclusions about the prognostic implications of micrometastases detected by OSNA. Finally, differences in surgical approaches among patients may have introduced potential selection bias. Despite these limitations, this study is the first to evaluate intraoperative whole SN assessment using the OSNA assay in cervical cancer, and it offers valuable preliminary insights into its potential clinical utility.

### 4.4. Implications for Practice and Future Research

This study demonstrated that intraoperative SN assessment using the OSNA assay in cervical cancer resulted in a higher detection rate of metastatic SNs than conventional pathological examination. Furthermore, the OSNA assay exhibits superior sensitivity in detecting micrometastases, which are often undetectable by standard histopathological evaluation. Additionally, our findings suggest that, in cases where SN metastases are limited to micrometastases, the likelihood of non-SN metastases may be considerably low. These results support the potential for a more conservative surgical approach in selected patients. However, to confirm these observations and guide clinical decision making, prospective multicenter studies with larger patient populations and long-term oncologic outcomes are strongly recommended.

## 5. Conclusions

This study demonstrated that intraoperative SN assessment using the OSNA assay in cervical cancer resulted in a higher detection rate of metastatic SNs than conventional pathological examination. The OSNA assay exhibited superior sensitivity in detecting micrometastases, which are often missed by standard histopathological techniques. Furthermore, the absence of non-SN metastasis in cases with only SN micrometastasis suggests the potential for omitting systematic lymphadenectomy in selected patients. These findings support the need for further prospective multicenter validation.

## Figures and Tables

**Table 1:** Clinicopathological characteristics.

	OSNA (n = 50)	Pathology (n = 113)	*p*-Value
Median age (years)	42 (29–78)	39 (20–74)	0.12
Median BMI (kg/m^2^)	21.9 (17.1–34.8)	21.6 (16.5–50.4)	0.40
Surgical procedure			0.0017
Laparotomy	20 (40%)	71 (63%)	
Laparoscopy	29 (58%)	33 (29%)	
Robot	1 (2%)	9 (8%)	
Final pathology			0.40
SCC	35 (70%)	82 (72%)	
Adenocarcinoma	12 (24%)	24 (21%)	
Adenosquamous cell carcinoma	0	4 (4%)	
Other	3 (6%)	3 (3%)	
Postoperative FIGO stage (2009)			0.0002
IA2	16 (32%)	20 (18%)	
IB1	12 (24%)	73 (65%)	
IB2	13 (26%)	14 (12%)	
IB3	1 (2%)	1 (1%)	
IIA1	2 (4%)	0	
IIB	0	1 (1%)	
IIIC1	6 (12%)	4 (3%)	
LVSI			0.02
No	26 (52%)	80 (71%)	
Yes	24 (48%)	33 (29%)	
Cervical stromal invasion			0.12
None	0	5 (5%)	
<1/2	36 (72%)	86 (76%)	
≥1/2	14 (28%)	22 (19%)	
Median number of SNs removed (range)	2 (2–7)	2 (1–6)	0.004
SN metastasis			0.038
Negative	44 (88%)	109 (97%)	
Positive	6 (12%)	4 (3%)	
Macrometastasis (++)	0	3	
Micrometastasis (+)	6	1	
Recurrence			0.18
No	50 (100%)	109 (96%)	
Yes	0	4 (4%)	
Adjuvant therapy			0.11
None	34 (68%)	92 (81%)	
Chemotherapy	4 (8%)	8 (7%)	
Radiotherapy	12 (24%)	13 (12%)	

BMI: body mass index, FIGO: International Federation of Gynecology and Obstetrics, LVSI: lymph–vascular space involvement, OSNA: one-step nucleic acid amplification.

**Table 2:** Clinicopathological analysis of patients with sentinel lymph node (SN) metastasis by the one-step nucleic acid amplification (OSNA) assay (**a**) and pathology (**b**).

**(a)**								
	**Patient 1**	**Patient 2**		**Patient 3**	**Patient 4**	**Patient 5**		**Patient 6**
Final pathology	SCC	SCC		Clear cell carcinoma	SCC	SCC		SCC
Surgical procedure	Laparotomy	Laparotomy		Laparoscopy	Laparoscopy	Laparotomy		Laparotomy
Cervical stromal invasion	1/2≤	1/2≤		<1/2	<1/2	<1/2		1/2≤
LVSI	Yes	No		Yes	No	Yes		No
SN location	External iliac	Obturator		External iliac	Obturator	External iliac		Obturator
Copy number (copies/μL)	5500 (+)	1700 (+)		1200 (+)	4100 (+)	3100 (+)		1700 (+)
Stamped cytology	Suspicious	Negative		Negative	Negative	Negative		Negative
Non-SN metastasis	No	No		No	No	No		No
Recurrence	No	No		No	No	No		No
**(b)**								
	**Patient 1**		**Patient 2**		**Patient 3**		**Patient 4**	
Final pathology	Adenosquamous cell carcinoma		SCC		SCC		SCC	
Surgical procedure	Laparoscopy		Laparotomy		Laparotomy		Laparotomy	
Cervical stromal invasion	1/2≤		<½		1/2≤		1/2≤	
LVSI	Yes		Yes		Yes		Yes	
SN location	External iliac		Obturator		External iliac		Obturator	
Non-SN metastasis	No		No		No		No	
Recurrence	No		No		No		No	

SCC: squamous cell carcinoma, LVSI: lymphovascular space involvement.

## Data Availability

The data presented in this study are available on reasonable request from the corresponding author.

## Abbreviations

The following abbreviations are used in this manuscript:

CK19	Cytokeratin 19
ICG	Indocyanine green
LVSI	Lymphovascular space invasion
OSNA	One-step nucleic acid amplification
PLA	Pelvic lymphadenectomy
SN	Sentinel lymph node
